# Association of Physical Activity Levels with Severe Multimorbidity Among Middle-Aged and Older Adults: Evidence from CHARLS and a Community Sample

**DOI:** 10.3390/healthcare14152417

**Published:** 2026-08-05

**Authors:** Shi-Han Liang, Ming-Yuan Zhao, Xiu-Han Zhao

**Affiliations:** 1 Graduate School, Harbin Sport University, Harbin 150096, China; 2 Faculty of Physical Education, Harbin Sport University, Harbin 150096, China; 3 College of Physical Education, Shandong University, Jinan 250102, China

**Keywords:** physical activity, severe multimorbidity, middle-aged and older adults, CHARLS, machine learning, burden stratification

## Abstract

**Background**: Multimorbidity imposes a substantial burden on older populations, yet research neglects how physical activity (PA) relates to severe disease burden in established cases. Moreover, the efficacy of combining low-cost PA assessments with routine indicators to identify high-burden patients in resource-constrained settings remains unclear. This study explores associations between PA levels and severe multimorbidity (≥4 conditions), evaluating PA’s utility for auxiliary status identification via interpretable machine learning. **Methods**: This cross-sectional study included the CHARLS 2018 sample (n = 10,130) and a supplementary community sample (n = 315). Multivariable logistic regression evaluated associations between PA and severe multimorbidity. An XGBoost model assessed routine indicators’ classification performance, utilizing SHAP values to analyze feature contributions to the model. **Results**: PA was inversely associated with severe multimorbidity specifically among those meeting the WHO criteria (MPA > 300 or VPA > 150 min/week) for additional health benefits (OR = 0.80, 95% CI: 0.73–0.88, *p* < 0.001), especially in adults ≥60 years. Although models using solely routine indicators showed modest discrimination (AUC = 0.673), SHAP analysis revealed PA as the foremost modifiable behavioral feature in classification, with local patterns aligning closely with regression findings. **Conclusions**: Among middle-aged and older adults with multimorbidity, PA levels exhibit significant categorical associations with severe multimorbidity. In community management, low-cost PA evaluation serves as an auxiliary indicator to preliminarily classify high-burden individuals. Given potential reverse causality, future longitudinal cohorts are required to clarify causal relationships, providing direct evidence for daily activity management.

## 1. Introduction

As global population aging accelerates, the disease burden is shifting from single chronic conditions toward a multimorbidity pattern [1,2]. Multimorbidity, commonly defined as the coexistence of two or more chronic diseases in an individual, is closely associated with high healthcare utilization, disability, mortality, and significantly reduced quality of life [3]. Data from the World Health Organization (WHO) indicate that, in a large systematic review of seven high-income countries, more than half of older adults were affected by multimorbidity, with prevalence rising sharply with age [4].

Against this backdrop, the population of middle-aged and older adults with multimorbidity is continuously expanding, and the number of coexisting chronic conditions per individual is steadily increasing [5]. While a simple disease count cannot fully capture the multidimensional clinical heterogeneity of multimorbidity, existing evidence suggests that the cumulative number of conditions is significantly associated with a heavier health burden. In particular, among older adults, using a cut-off of four or more chronic conditions more effectively identifies those with a high disease burden [6]. Therefore, based on this cut-off, exploring factors associated with the characteristics of high-burden populations is of great significance for improving primary public health prevention and control strategies.

Physical activity (PA) is a key modifiable behavior in chronic disease management for middle-aged and older adults. Regular PA enhances cardiorespiratory fitness, metabolic status, and muscle strength, and is associated with a lower multimorbidity burden [7,8,9]. However, previous studies have largely been confined to a single-disease prevention perspective or have treated multimorbidity as a simple binary outcome (presence vs. absence), focusing more on primary prevention in the general population [10]. In fact, one of the challenges in primary care is the effective management of individuals who already have chronic conditions [11]. Analyzing healthy individuals, those with a single chronic disease, and those with multimorbidity together may mask characteristic differences within the high-burden subgroup of the multimorbidity population [12]. Therefore, this study explicitly focuses on middle-aged and older adults who have already been diagnosed with multimorbidity (≥2 conditions) and examines the association between different PA levels and the state of severe multimorbidity (≥4 conditions).

Elucidating this association would not only deepen our understanding of differences in the severity of multimorbidity, but also offer a potential entry point for identifying individuals with a high disease burden in primary care settings. Currently, community-based health management often has access only to routine indicators such as demographic and lifestyle information and lacks in-depth clinical data, making it difficult to accurately identify individuals with severe multimorbidity [13]. Although low-cost PA assessment provides a simple tool for preliminary identification in primary care, given that the identification and management of multimorbidity are constrained by multidimensional factors, a single indicator is insufficient to serve this purpose independently [14,15]. Therefore, objectively evaluating the combined classification performance of these routinely available indicators and clarifying the relative importance of PA within them could effectively overcome clinical and resource barriers, which has practical significance for primary public health practice.

To address these needs, machine learning offers a promising approach for handling complex health data. Compared with conventional statistical models, machine learning demonstrates marked advantages in capturing nonlinear relationships and higher-order interaction effects [16]. In particular, explainable machine learning can enhance the interpretability of results without compromising model discrimination. Previous studies have already demonstrated its potential for identifying key associated factors in exercise rehabilitation and functional assessment [17,18].

Accordingly, the primary objective of this study is to examine the association between PA levels and severe multimorbidity (≥4 conditions) among middle-aged and older adults with multimorbidity (≥2 conditions). The secondary objective is to evaluate the feasibility of developing a machine learning-based auxiliary identification model for severe multimorbidity using routinely available community indicators, with a specific focus on analyzing the feature contribution of PA in this identification process. The findings are expected to provide evidence for burden stratification, individualized physical activity regimens, and the optimization of primary health management strategies for populations with severe multimorbidity.

## 2. Materials and Methods

### 2.1. Data Source

This cross-sectional study utilized data from the 2018 China Health and Retirement Longitudinal Study (CHARLS), a nationally representative survey of Chinese residents aged 45 and older that employed a multi-stage stratified probability sampling design to collect multidimensional health and socioeconomic information. CHARLS has been widely utilized in health research among middle-aged and older adults [19]. To avoid selection bias, we retained the original CHARLS target population, restricting our primary sample to respondents aged ≥45 years. The CHARLS project was approved by the Biomedical Ethics Committee of Peking University (IRB00001052-11015), and all participants provided written informed consent. The data had been de-identified prior to public release, and our analyses followed the relevant data use agreements.

To examine the applicability of the core association patterns in real-world community settings, we additionally drew on data from a questionnaire survey conducted in May 2026 across eight communities in China. This survey adopted a community-based random sampling strategy. Given that adults aged ≥60 years represent the primary demographic for primary public health and chronic disease management, and considering daytime sampling constraints at community health centres (which largely precluded the recruitment of employed individuals aged 45–59), this sample was restricted to older adults. Accordingly, the inclusion criteria for the community sample were: (1) age ≥ 60 years; (2) continuous local residence for at least 6 months; (3) normal cognitive function and no communication barriers; (4) voluntary participation with signed informed consent. Exclusion criteria comprised severe physical illness, severe visual or hearing impairment, or the inability to cooperate with the questionnaire. All data were collected by standardized trained interviewers via face-to-face interviews, under strict quality control protocols throughout the process. After verification, the data were immediately de-identified and stored in encrypted form, accessible only to authorised researchers. The informed consent obtained from participants covered full authorisation for questionnaire participation, data de-identification, and subsequent secondary academic analyses. This community-based investigation was approved by the Academic Ethics Committee of Harbin Sport University (Approval No. 2026054). Data cleaning, variable construction, and statistical analyses in this study were performed using Stata 18.0 and Python 3.13 environments.

### 2.2. Study Population

**Figure 1:**
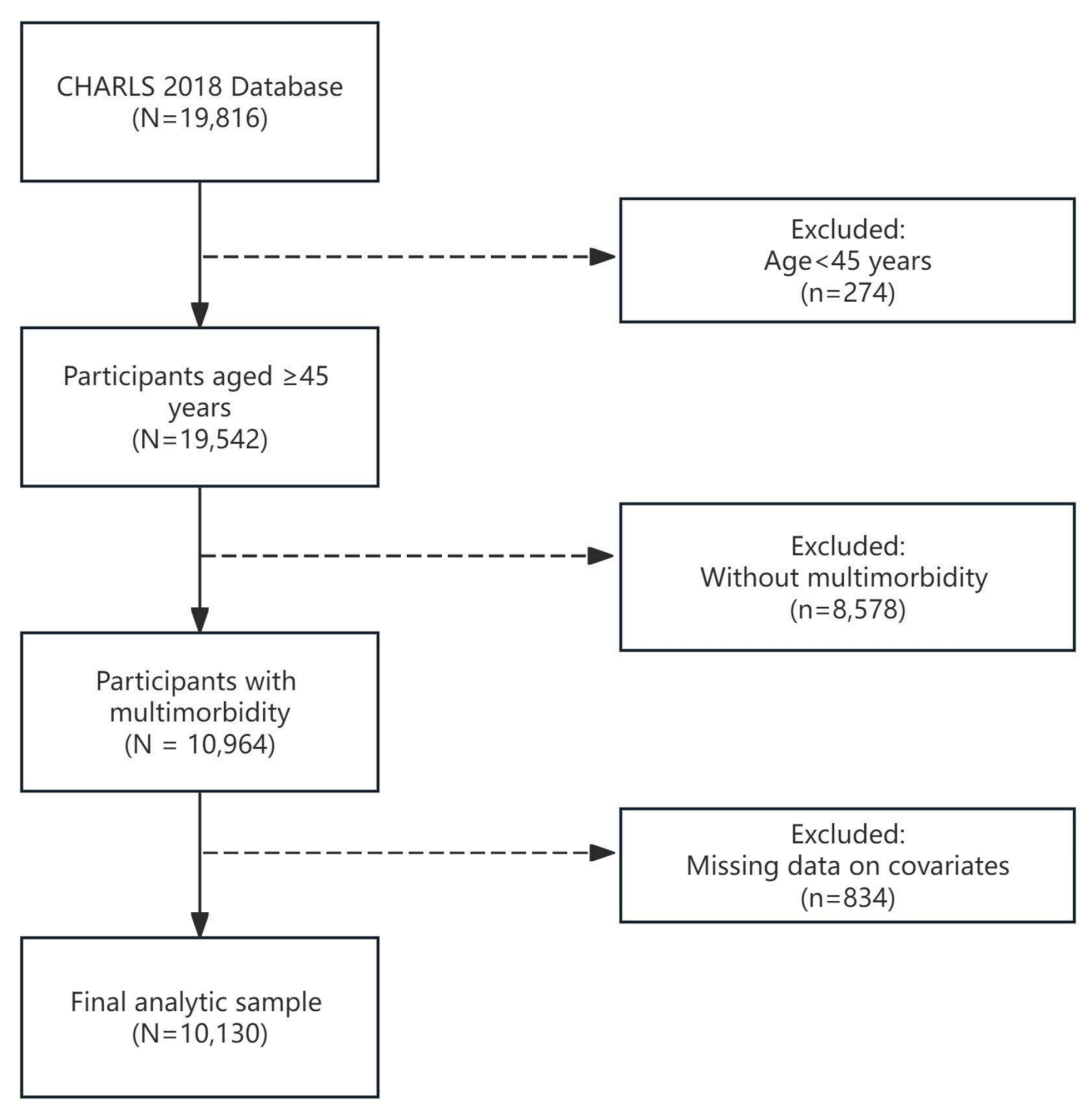
Flowchart of the Participant Selection Process.

From the 2018 CHARLS database, the initial sample comprised 19,816 respondents. A stepwise screening process was implemented according to the study objectives and predefined criteria: we first excluded those aged <45 years (n = 274), then removed participants who did not meet the multimorbidity criterion (fewer than two chronic conditions, n = 8578), and finally excluded those with incomplete key covariate data (n = 834). This yielded a final primary analysis sample of 10,130 respondents aged ≥45 years with multimorbidity (Figure 1).

For the community sample, 400 questionnaires were distributed, yielding 392 valid responses (response rate 98.0%). After further excluding those who did not meet the multimorbidity criteria (n = 77), 315 older adults aged ≥60 years with multimorbidity were included.

Notably, the two datasets differ inherently in age structure, timing of data collection, dimensions of chronic disease measurement, and survey contexts; therefore, the community sample is not an independently replicated cohort fully matched to CHARLS. Accordingly, we explicitly designated CHARLS as the primary analysis dataset for conducting the core association analyses and machine learning modelling. The community data, in turn, serve as a supplementary sample, intended to examine whether the direction of associations and core patterns identified in the main dataset can be effectively observed in a real-world community-dwelling older population, rather than to directly compare prevalence rates or absolute magnitudes of effect estimates between the two samples.

### 2.3. Variables

#### 2.3.1. Outcome Variable

This study assessed 14 chronic conditions covered in the CHARLS database, including hypertension, diabetes, malignant neoplasms, liver diseases, kidney diseases, stomach diseases, asthma, chronic lung diseases, heart disease, stroke, psychiatric disorders, arthritis, dyslipidemia, and memory impairment. A count-based approach was used to grade the severity of multimorbidity. Following established criteria, we defined the presence of 2–3 chronic conditions as “common multimorbidity” and ≥4 conditions as “severe multimorbidity” [20,21].

Of note, although this grouping facilitates stable and reproducible stratification in large populations and helps identify individuals with a higher disease burden and a greater number of coexisting adverse health conditions, disease count alone cannot fully capture clinical severity, disease–disease interactions, frailty status, or functional impairment [22,23]. Therefore, the term “severe multimorbidity” herein refers specifically to a state of high disease burden defined by a numeric threshold, rather than clinical severity per se. Detailed grouping criteria are provided in the Supplementary Material (Supplementary Table S1).

For the community sample, to balance survey feasibility and cross-sample comparability, we selected seven core chronic conditions that matched those in CHARLS (hypertension, dyslipidemia, diabetes, heart disease, stroke, memory impairment, and psychiatric disorders) and retained the cutoff of ≥4 conditions for defining severe multimorbidity.

#### 2.3.2. Exposure Variable

Physical activity (PA) level was selected as the primary exposure variable. From the CHARLS dataset, we extracted categorical information on the frequency and duration per session of moderate-intensity (MPA) and vigorous-intensity (VPA) physical activity per week, and determined the PA level based on predefined rules. Given that the original CHARLS questionnaire collected PA duration in categorical intervals rather than exact continuous minutes, further calculating weekly equivalent total volume would require assigning representative values to each interval and making additional assumptions. Therefore, in the primary analysis, we did not directly use a continuous volume measure combining moderate-to-vigorous PA; instead, we constructed a rule-based three-category PA variable. Based on the thresholds recommended in the WHO 2020 guidelines on physical activity and sedentary behaviour [24], we classified PA into three levels: (1) inactive (MPA < 150 min/week and VPA < 75 min/week); (2) active (MPA 150–300 min/week or VPA 75–150 min/week), i.e., meeting the basic PA recommendation; (3) highly active (MPA > 300 min/week or VPA > 150 min/week), i.e., meeting the higher threshold for additional health benefits. The three levels were defined using hierarchical mutually exclusive criteria: “highly active” was assigned first, followed by “active” among those who did not meet the highly active criteria, and the remainder were classified as “inactive”. Detailed variable construction methods are provided in the Supplementary Material (Supplementary Table S2).

Of note, in line with WHO guideline principles, PA in this study is not restricted to structured exercise but encompasses the total volume of all activities in daily life that meet the corresponding intensity criteria, including leisure-time exercise, occupational labour, housework, and transportation.

For the independent community sample, PA information was collected following the CHARLS and WHO principles, with considerations for field survey feasibility. To ensure data comparability, we retained the same three-category classification strategy. Given that detailed collection of activity at different intensities might increase the cognitive burden on older respondents, the questionnaire adopted a single-choice design based on the highest intensity level. Specific questionnaire items and classification criteria are available in the Supplementary Material (Supplementary Table S3).

#### 2.3.3. Covariates

Covariate selection was guided by prior literature and data availability [25,26], and strictly followed the principle of “routine, low-cost, and easily accessible” in primary care settings, aiming to construct a parsimonious classification scenario for high-burden status that aligns with real-world needs. Based on this, the covariates included in the analyses were classified into three categories: (1) demographic characteristics, including age, sex, marital status, and educational attainment; (2) health-related behaviors, including smoking and drinking status; (3) health status measures, including self-rated health (SRH) and functional impairment in activities of daily living (ADL). Notably, SRH and ADL are closely related to multimorbidity severity; they could serve as potential confounders reflecting baseline health differences related to both PA and multimorbidity, or they might lie on the pathway between disease burden, physical function, and activity behaviour, thereby possibly acting as mediators or sources of overadjustment bias [26,27]. Consequently, our main models adjusted only for demographic characteristics and health behaviours, whereas the sensitivity analyses further included SRH and ADL to evaluate the robustness of the primary associations after additional control for baseline health and functional status. Specific variable coding is detailed in the Supplementary Material (Supplementary Table S4).

### 2.4. Statistical Analysis

Baseline characteristics of the participants were summarized using descriptive statistics. Continuous data approximating a normal distribution were expressed as mean ± standard deviation, and compared via one-way analysis of variance (ANOVA). Categorical variables were presented as frequencies and percentages, with differences across groups evaluated using the Pearson chi-square test [28].

Subsequently, multivariable logistic regression models were constructed to evaluate the association between different PA levels and severe multimorbidity. Effect sizes were presented as odds ratios (ORs) alongside 95% confidence intervals (CIs). We set up three regression models step by step to control for confounders. The first model contained no adjusted variables. The second model accounted for demographic factors such as age, sex, marital status and educational attainment. On the basis of Model 2, Model 3 additionally included smoking and drinking status. Subsequently, sensitivity analyses were performed by further incorporating SRH and ADL indicators into Model 3 to verify the robustness of the main findings.

Subgroup analyses were stratified by sex and age (<60 vs. ≥60 years) to explore potential heterogeneous effects. To examine interaction effects, interaction terms between PA and the stratifying variables were introduced into the regression models, and the Wald test was applied for significance evaluation. All subgroup models used the same adjusted factors as Model 3, excluding the stratifying variable itself.

Furthermore, to verify the reliability of the core conclusions and address potential methodological bias, we conducted the following three supplementary and sensitivity analyses using the same regression strategy as the main analysis: (1) full-population regression analysis: to assess the potential selection bias introduced by restricting the main analysis to individuals already affected by multimorbidity (≥2 conditions), we re-included the full age-eligible population with 0–1 chronic conditions into the model to observe whether the association changed in the whole sample (Supplementary Table S5); (2) PA classification replacement: to rule out the influence of the chosen classification thresholds on the conclusions, we recalculated PA using a continuous volume method that combined moderate-to-vigorous activity (with 1 min of VPA equivalent to 2 min of MPA) following WHO guidelines, and repeated the association analysis (Supplementary Table S6); (3) outcome definition matching: given that the community sample covered only seven core chronic conditions, to reduce discrepancies in outcome measurement dimensions, we re-defined severe multimorbidity (≥4 conditions) in the main CHARLS sample using only these seven common diseases and repeated the analysis (Supplementary Table S7).

We used two-sided tests for all statistical analyses, and a *p*-value below 0.05 was defined as statistically significant. All data processing and statistical analyses were executed using Stata 18.0.

### 2.5. Machine Learning Modelling and Interpretability Analysis

To complement conventional regression analyses, we introduced machine learning methods to develop a model for identifying individuals with severe multimorbidity, and to explore complex patterns and interaction structures among the variables. After handling missing data via listwise deletion, the 10,130 participants in the primary sample were randomly partitioned into training, test, and validation sets at a ratio of 7:1.5:1.5. Following the modelling frameworks of previous comparable machine learning studies, we constructed six supervised learning algorithms in parallel, namely LightGBM, Logistic Regression (LR), Multilayer Perceptron (MLP), Random Forest (RF), Support Vector Machine (SVM), and XGBoost [29,30]. For each algorithm, we adopted empirical hyperparameter settings and controlled overfitting by monitoring performance on the validation set. Meanwhile, given the importance of preserving the true prevalence distribution and ensuring model calibration, we did not apply any class imbalance handling strategies such as resampling or class weighting. Model performance was evaluated using AUC, accuracy, F1 score, precision, sensitivity, and specificity, supplemented by ROC curves, calibration curves, Precision–Recall curves, and decision curve analysis to assess practical utility [31].

After selecting the model with the best generalisation performance, we applied the SHAP (SHapley Additive exPlanations) method to further quantify the relative contributions of PA and each covariate to the model classification, and to visualise their association patterns with the model predictions. All machine learning modelling and interpretability analyses were performed in Python 3.13.

## 3. Results

### 3.1. Baseline Characteristics of the Study Participants

A total of 10,130 middle-aged and older adults with multimorbidity (aged ≥ 45 years) were included in the final analysis (Table 1). The overall mean age was 63.49 ± 9.46 years; 4590 participants (45.3%) were male and 5540 (54.7%) were female. Of these, 3867 (38.2%) met the criteria for severe multimorbidity. When categorized by PA levels, the highly active group comprised 4040 individuals (39.9%), the active group 916 (9.0%), and the inactive group 5174 (51.1%).

Baseline comparisons revealed statistically significant differences in all characteristics across PA groups (all *p* < 0.001). Moreover, as PA levels declined, the proportion of individuals with severe multimorbidity exhibited a stepwise increase (33.5% in the highly active group vs. 38.9% in the active group vs. 41.7% in the inactive group). Baseline characteristics of the Supplementary community sample are also detailed in the Supplementary Material (Supplementary Table S8).

### 3.2. Association Between Physical Activity and Severe Multimorbidity

#### 3.2.1. Regression Results in the Primary Sample

Using the inactive group as the reference, multivariable logistic regression demonstrated that the highly active group was consistently associated with lower odds of severe multimorbidity in the CHARLS sample (Table 2).

Specifically, in the unadjusted model, the highly active group was associated with lower odds of severe multimorbidity (OR = 0.71, 95% CI: 0.65–0.77, *p* < 0.001). Upon adjustment for age, sex, marital status, and educational attainment, this association was slightly attenuated, and remained virtually unchanged in the primary fully adjusted model after further adjusting for smoking and drinking status (OR = 0.80, 95% CI: 0.73–0.88, *p* < 0.001).

In sensitivity analyses, after additional adjustment for SRH and ADL, the association between the highly active group and severe multimorbidity was further attenuated but remained statistically significant (OR = 0.89, 95% CI: 0.81–0.98, *p* = 0.015). Conversely, no statistically significant associations were observed for the active group in any of the four models (all *p* > 0.05).

#### 3.2.2. Subgroup Analyses

Sex-stratified subgroup analyses revealed that the association pattern between PA and severe multimorbidity was consistent between men and women (Table 3). Specifically, the highly active group was significantly associated with lower odds of severe multimorbidity in both women (OR = 0.78, 95% CI: 0.69–0.88, *p* < 0.001) and men (OR = 0.83, 95% CI: 0.73–0.95, *p* = 0.006), whereas the active group showed no statistically significant association in either sex. Interaction tests further indicated no significant interaction between PA and sex (*p* for interaction = 0.828), suggesting an absence of clear sex heterogeneity (Table 4).

By contrast, age-stratified subgroup analyses showed that age may have a modifying effect on the aforementioned association (Table 5). Among participants aged ≥60 years, the highly active group was significantly associated with lower odds of severe multimorbidity (OR = 0.71, 95% CI: 0.64–0.80, *p* < 0.001), whereas the active group showed a marginal, though non-significant, association (OR = 0.85, 95% CI: 0.73–1.01, *p* = 0.073). However, in those aged <60 years, neither the active nor the highly active group demonstrated a statistically significant association. Further interaction tests revealed that the interaction term between PA and age group was statistically significant (*p* for interaction = 0.025), indicating that the inverse association between highly active PA and severe multimorbidity was more pronounced in the older (≥60 years) demographic (Table 4).

#### 3.2.3. Community Sample and Sensitivity Analyses

Analyses in the supplementary community sample showed that the direction of associations was highly consistent with that in the CHARLS primary sample (Table 6). In the unadjusted model, the highly active group was also significantly associated with lower odds of severe multimorbidity (OR = 0.28, 95% CI: 0.14–0.54, *p* < 0.001), and after adjusting for all covariates, this association remained robust (OR = 0.21, 95% CI: 0.09–0.49, *p* < 0.001). Consistent with the primary sample, the active group showed no significant association in any model within the community sample.

Moreover, the three sensitivity analyses conducted in this study further supported the above findings. Whether in the full-population regression analysis that included individuals with 0–1 chronic conditions (Supplementary Table S5), in the reclassification of PA using the WHO equivalent activity volume (Supplementary Table S6), or in the redefinition of the outcome based on the seven common diseases in the CHARLS primary sample (Supplementary Table S7), the significant association between the highly active group and lower odds of severe multimorbidity remained robust across all models under these alternative specifications, further supporting the reliability of the core conclusions.

### 3.3. Machine Learning Model Analysis

#### 3.3.1. Evaluation of Model Identification Performance

**Figure 2:**
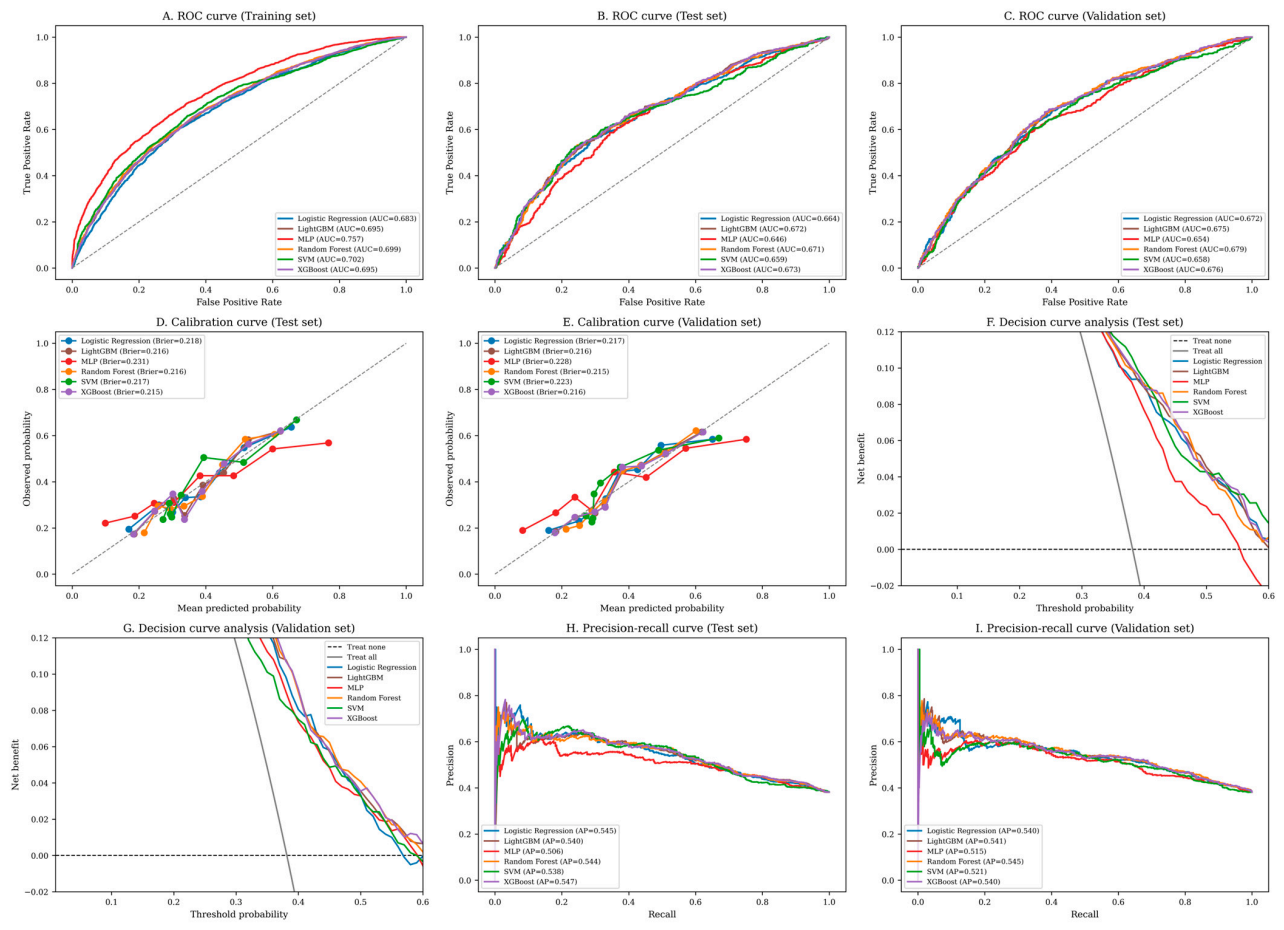
Performance Comparison of Six Predictive Models (**A**) ROC curves for the training set; (**B**) ROC curves for the test set; (**C**) ROC curves for the validation set; (**D**) calibration curves for the test set; (**E**) calibration curves for the validation set; (**F**) decision curve analysis for the test set; (**G**) decision curve analysis for the validation set; (**H**) precision–recall curves for the test set; and (**I**) precision–recall curves for the validation set.

The performance of six machine learning models was evaluated for identifying severe multimorbidity (Table 7, Figure 2). Overall, the discriminative ability of all models was modest. Accuracy on the test and validation sets ranged from 64% to 66%, and F1 scores were relatively low, between 0.38 and 0.47. All models exhibited high specificity but low sensitivity: specificity generally ranged from 78.6% to 88.3%, whereas sensitivity ranged only from 27.8% to 40.9%. Decision curve analysis (Figure 2F,G) further suggested that, although the models could reasonably rule out low-burden individuals, their ability to identify high-burden cases was limited, yielding a narrow range of net benefit.

Among the individual algorithms, MLP achieved the highest AUC on the training set (AUC = 0.757), but its performance dropped markedly on the test set (AUC = 0.646) and validation set (AUC = 0.654), indicating substantial overfitting. In contrast, tree-based ensemble methods (LightGBM, Random Forest, and XGBoost) generalised better, with AUC values consistently around 0.67 on both the test and validation sets. Among these, Random Forest performed relatively well on the validation set (AUC = 0.679), though it decreased slightly on the test set (AUC = 0.671). LightGBM and XGBoost demonstrated comparable overall performance, but XGBoost showed marginally higher AUC values on both the test set (0.673 vs. 0.672) and validation set (0.676 vs. 0.675). Calibration curves (Figure 2D,E) also indicated that XGBoost achieved the optimal or tied-for-optimal predicted probability agreement on the test set (Brier = 0.215) and validation set (Brier = 0.216).

Consequently, considering the advantages in both generalisation stability and calibration performance, XGBoost was selected for the subsequent SHAP-based interpretability analysis.

#### 3.3.2. SHAP-Based Interpretability Analysis

Based on the optimal XGBoost model, SHAP values were extracted to quantify the overall contribution of each feature to the model’s classification decisions and their association patterns. A positive SHAP value indicated that the feature was associated with a higher probability of severe multimorbidity, whereas a negative value indicated the opposite.

**Figure 3:**
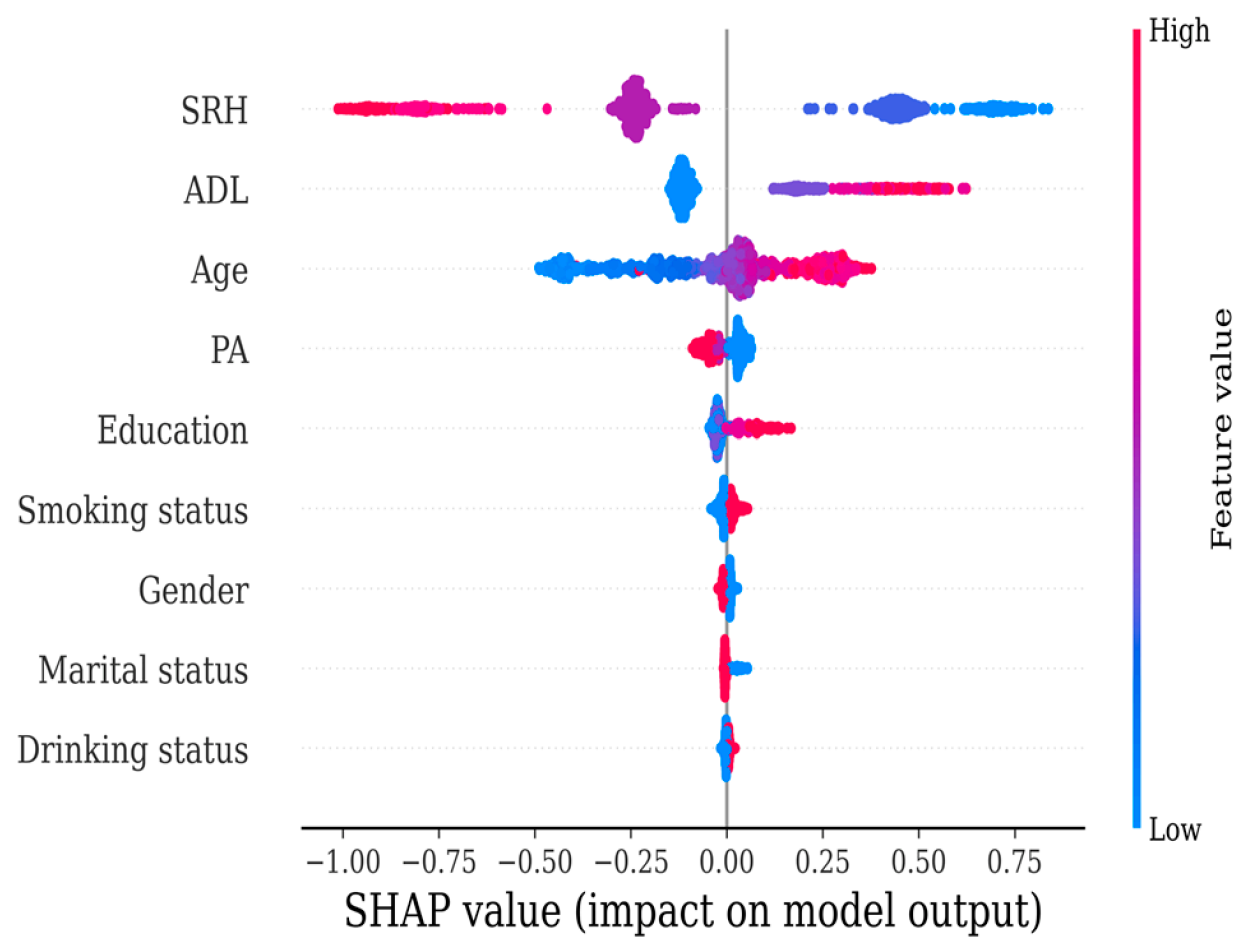
SHAP Summary Plot Showing the Distribution and Direction of Feature Contributions to Model Prediction.

First, the global SHAP summary plot (Figure 3) revealed that SRH, ADL, and age were the core features contributing most to the model output. PA ranked immediately after them as the highest-ranking modifiable behavioural variable, whereas educational attainment, smoking, and drinking status contributed relatively weakly.

**Figure 4:**
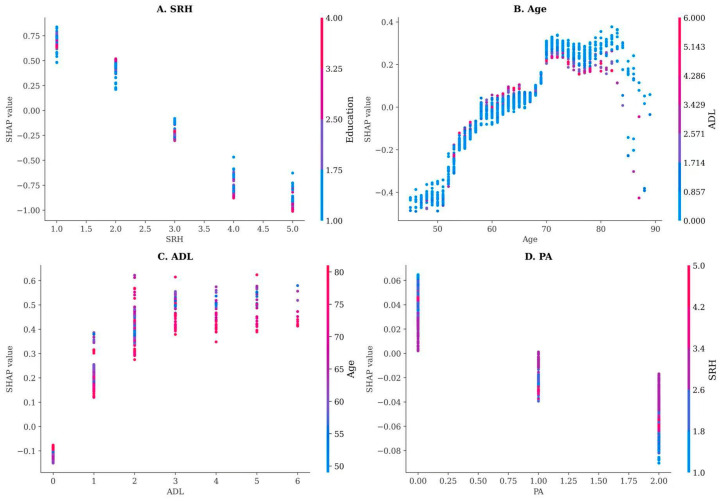
SHAP Dependence Plots for Key Predictors (**A**) Self-rated health (SRH); (**B**) Age; (**C**) Activities of daily living (ADL); (**D**) Physical activity (PA).

Second, SHAP dependence plots (Figure 4) further illustrated the associations and feature interactions among the core variables. For SRH, poorer health status corresponded to higher positive SHAP contributions, and this pattern was more pronounced among individuals with lower educational attainment. For ADL, transitioning from “no functional limitation” (0) to “any mild impairment” (1) produced an abrupt step-like jump in probability contribution. Age interacted with ADL, and its SHAP values shifted from negative to positive around age 60, reaching a plateau between 70 and 80 years. Regarding PA, only the highly active group consistently exhibited negative contributions, whereas the inactive group showed substantially positive contributions, and the active group fluctuated around zero. In addition, a notable interaction between PA and SRH was observed, with the combination of “inactive” and “very poor health” yielding the highest positive contribution.

**Figure 5:**
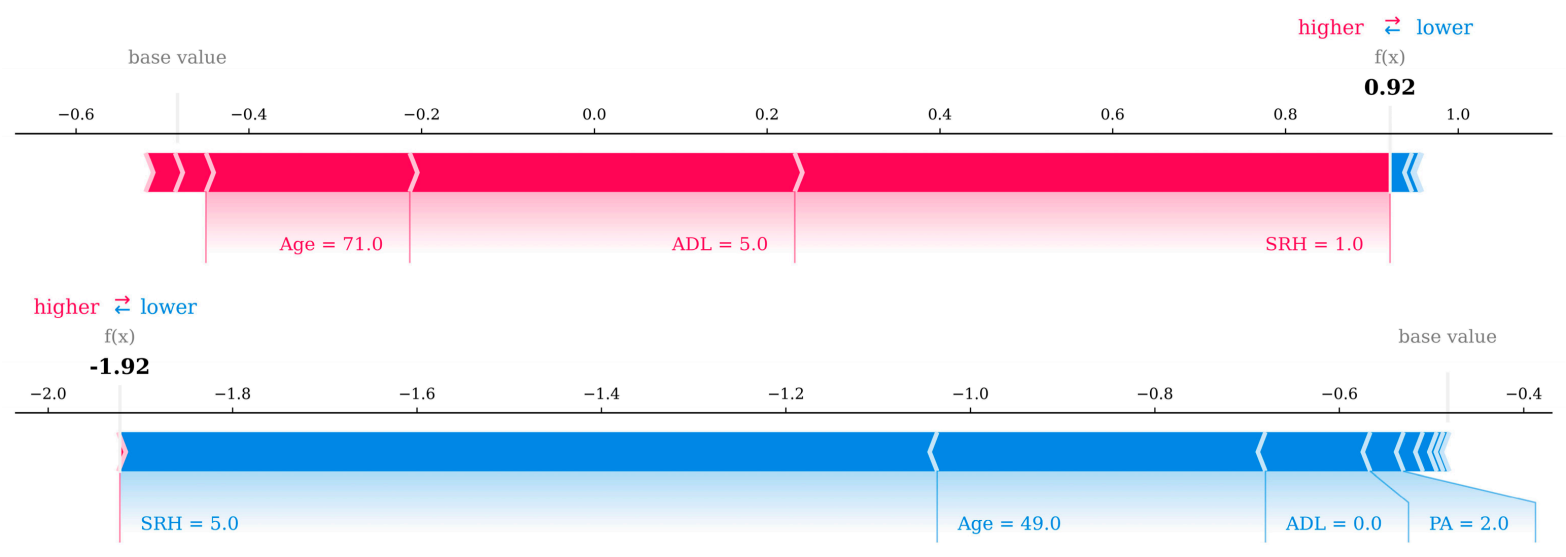
SHAP Force Plots for Representative Individuals with High and Low Probabilities of Severe Multimorbidity.

Finally, SHAP force plots (Figure 5) translated the above global patterns to the individual level. The results revealed that even among individuals with similar total probability scores, the combinations of contributing features varied considerably across participants, underscoring the complexity inherent in the classification pattern of multimorbidity.

## 4. Discussion

### 4.1. Stratified Association Pattern Between Physical Activity and Severe Multimorbidity

Unlike previous studies that focused primarily on the presence or absence of disease, we concentrated on the differentiation of disease burden within the already-affected population. Our results demonstrated a pattern of stratified associations between PA and severe multimorbidity: a significant association with lower disease burden was observed only when PA reached the WHO criteria for additional health benefits (MPA > 300 min/week or VPA > 150 min/week), whereas the group meeting only the basic recommendation (MPA 150–300 min/week or VPA 75–150 min/week) did not differ significantly from the inactive group. This pattern remained robust after adjustment for confounders, in the supplementary community sample, and in sensitivity analyses. Subgroup analyses further suggested that this association pattern was more pronounced among individuals aged ≥60 years.

This may indicate that, as age advances and physiological reserves decline, higher total activity volume could be associated with the maintenance of better health status through shared pathways such as improved cardiorespiratory and metabolic fitness and reduced chronic inflammation [32,33], implying that PA may serve as an important behavioural pillar for maintaining health in older adults. However, this should not be interpreted as endorsing indiscriminate high-volume exercise for middle-aged and older adults with multimorbidity. Recent evidence has indicated that repeated excessive PA loads can induce synovial inflammation and suppress chondrocyte function, thereby accelerating joint degeneration—a concern particularly relevant to multimorbid individuals with coexisting musculoskeletal conditions [34]. Moreover, given the inherent limitations of the cross-sectional design, the observed associations may also stem from reverse causality, whereby individuals with a higher disease burden objectively find it more difficult to maintain high activity levels. Therefore, in public health practice, safety and tolerability must be the priority rather than blindly encouraging increased activity.

Under this premise, the lack of a statistically significant association for the group meeting only the basic PA recommendation does not imply an absence of health value. This might be attributable to the baseline characteristics of our study population. The WHO basic recommendation targets primary prevention in the general population, whereas in middle-aged and older adults with established multimorbidity, pathological damage has already accumulated. At this stage, basic activity levels may primarily contribute to symptom relief and quality-of-life improvement—effects that are unlikely to be captured by differences in disease counts in cross-sectional data.

In addition, based on the intention of constructing a “low-cost classification scenario”, we did not include indicators such as socioeconomic status or body mass index in the models. However, our PA measure encompassed occupational and daily life activities, and such non-leisure activities are often closely intertwined with an individual’s socioeconomic background. Therefore, limited by the inability to fully control for these potential confounders and the inherent constraints of the cross-sectional design, we cannot determine whether higher PA independently affects the accumulation process of chronic conditions. Future high-quality large-scale longitudinal cohort studies, with comprehensive adjustment for confounders and repeated dynamic measurements, are warranted to further clarify the causal direction between PA and disease accumulation, thereby providing a more solid evidence base for exercise prescription in populations with multimorbidity.

### 4.2. Machine Learning Performance Evaluation and Exploratory Analysis of Physical Activity Characteristics

Building on conventional regression models, we introduced explainable machine learning (SHAP) to evaluate the comprehensive classification capacity of routinely available community indicators—including PA—for severe multimorbidity. Our results showed that even with the XGBoost algorithm, which demonstrated favourable generalisation, the overall discriminative performance of the model based on routine variables remained modest (AUC = 0.673, Sensitivity = 35.52%). This indirectly reflects the nature of severe multimorbidity as a complex systemic syndrome that results not from the simple addition of individual behavioural or demographic factors, but from the long-term accumulation of multidimensional physiological impairments and pathological processes [35]. It also suggests that relying solely on brief questionnaires and superficial variables may be insufficient for accurate identification at the cross-sectional level. Future development of high-precision stratification tools for individuals with severe multimorbidity will need to incorporate deeper clinical data, including disease duration, biomarkers, and medication burden [36].

Despite the limited overall classification performance, the feature importance analysis derived from the machine learning models provided a quantitative perspective on the contribution of PA to the classification. The global SHAP analysis identified SRH, ADL, and age as the core contributors to model classification, with PA ranking immediately after them and being the top modifiable behavioural variable. More importantly, the contribution pattern of PA to model output, as shown in the SHAP dependence plots, was highly consistent with the association pattern observed in regression analyses.

Based on the above findings, the overall discriminative capacity of the current model based on routine variables is limited. Therefore, it should be described as an exploratory contemporaneous classification model rather than a validated clinical screening tool. Nevertheless, PA assessment is low-cost and easy to implement in the community, and it consistently demonstrated a stable contribution in our study. In real-world primary care settings with limited resources, although PA alone cannot serve as a standalone diagnostic tool, its stable contribution suggests it could serve as an auxiliary identification feature. It could provide extra behavioural information to assist primary health workers in preliminarily identifying and specifically focusing on individuals with a higher likelihood of severe multimorbidity. However, whether this application can be extended to clinical diagnosis or prognostic evaluation requires further research validation.

### 4.3. Limitations

Several limitations of this study should be acknowledged. First, the cross-sectional design precludes causal inference, and we cannot rule out reverse causality—that is, individuals with more severe disease may have reduced PA due to physical limitations; moreover, restricting the target population to those already diagnosed with multimorbidity inevitably introduces selection bias. Second, the core variables were largely derived from self-reports, which introduces recall bias; in addition, a simple disease count cannot fully capture the clinical severity and complexity of different disease combinations. Third, limited by the questionnaire structure, we adopted a conservative PA classification strategy, without precisely calculating the equivalent total activity volume or distinguishing between occupational and leisure-time activities. Fourth, the supplementary community sample had a relatively limited sample size and differed from the primary sample in survey context; thus, caution is warranted when extrapolating the effect estimates to other settings. Fifth, the overall classification accuracy of the machine learning model constructed in this study was modest and has not yet met the performance standards for independent clinical application. Future studies will need to incorporate multidimensional deep clinical data to further enhance its classification performance.

## 5. Conclusions

Using the nationally representative 2018 CHARLS sample (n = 10,130) and a supplementary community replication sample (n = 315), this study indicated a differential association pattern between PA level and severe multimorbidity (≥4 conditions) among middle-aged and older adults with multimorbidity. A significant inverse association with lower odds of severe multimorbidity was observed only when PA reached the WHO criteria for additional health benefits (MPA > 300 min/week or VPA > 150 min/week), and this inverse association was more pronounced in those aged ≥60 years. Furthermore, machine learning analyses revealed that, although the inherent complexity of severe multimorbidity limited the overall accuracy of models based on routine community indicators, PA remained the highest-ranking modifiable behavioural contributor to model classification, with its contribution pattern closely aligning with the regression results. These findings suggest that in community-based chronic disease management, low-cost PA assessment can serve as an auxiliary indicator to preliminarily classify individuals with a high disease burden and help primary care workers better understand an individual’s health burden status. Given the cross-sectional nature of this study, future large-scale prospective longitudinal cohorts are needed to further elucidate the causal associations between different PA doses and the trajectory of multimorbidity progression, thereby providing direct evidence for the development of physical activity regimens for affected populations.

## Figures and Tables

**Table 1:** Baseline Characteristics of the Study Participants in CHARLS.

Variable	Category	Highly Active (N = 4040)	Active (N = 916)	Inactive (N = 5174)	Total (N = 10,130)	*p-Value*
Age, Mean ± SD		60.65 ± 8.50	63.22 ± 8.88	65.75 ± 9.67	63.49 ± 9.46	*<0.001*
Gender, n (%)	Female	2130 (52.7%)	556 (60.7%)	2854 (55.2%)	5540(54.7%)	*<0.001*
	Male	1910 (47.3%)	360 (39.3%)	2320 (44.8%)	4590(45.3%)	
Marital status, n (%)	No	453 (11.2%)	138 (15.1%)	1028 (19.9%)	1619(16.0%)	*<0.001*
	Yes	3587 (88.8%)	778 (84.9%)	4146 (80.1%)	8511(84.0%)	
SRH, n (%)	Very poor	283 (7.0%)	66 (7.2%)	572 (11.1%)	921 (9.1%)	*<0.001*
	Poor	1073 (26.6%)	255 (27.8%)	1614 (31.2%)	2942(29.0%)	
	Fair	2114 (52.3%)	458 (50.0%)	2307 (44.6%)	4879(48.2%)	
	Good	324 (8.0%)	79 (8.6%)	402 (7.8%)	805 (8.0%)	
	Very good	246 (6.1%)	58 (6.3%)	279 (5.4%)	583 (5.7%)	
ADL, n (%)	No	3265 (80.8%)	737 (80.5%)	3586 (69.3%)	7588(74.9%)	*<0.001*
	1	440 (10.9%)	95 (10.4%)	677 (13.1%)	1212(12.0%)	
	2	153 (3.8%)	37 (4.0%)	366 (7.1%)	556 (5.5%)	
	3	91 (2.3%)	26 (2.8%)	204 (3.9%)	321 (3.2%)	
	4	51 (1.3%)	11 (1.2%)	152 (2.9%)	214 (2.1%)	
	5	28 (0.7%)	6 (0.7%)	124 (2.4%)	158 (1.6%)	
	6	12 (0.3%)	4 (0.4%)	65 (1.3%)	81 (0.7%)	
Drinking, n (%)	NO	2020 (50.0%)	496 (54.1%)	2875 (55.6%)	5391(53.2%)	*<0.001*
	YES	2020 (50.0%)	420 (45.9%)	2299 (44.4%)	4739(46.8%)	
Smoking, n (%)	NO	2301 (57.0%)	582 (63.5%)	2996 (57.9%)	5879(58.0%)	*<0.001*
	YES	1739 (43.0%)	334 (36.5%)	2178 (42.1%)	4251(42.0%)	
Education, n (%)	Primary school or below	1645 (40.7%)	357 (39.0%)	2261 (43.7%)	4263(42.1%)	*<0.001*
	Junior high school	1200 (29.7%)	255 (27.8%)	1330 (25.7%)	2785(27.5%)	
	Senior high/vocational	796 (19.7%)	175 (19.1%)	955 (18.5%)	1926(19.0%)	
	College or above	399 (9.9%)	129 (14.1%)	628 (12.1%)	1156(11.4%)	
Multimorbidity Count, n (%)	2	1598 (39.6%)	305 (33.3%)	1735 (33.5%)	3638 (35.9%)	*<0.001*
	3	1087 (26.9%)	255 (27.8%)	1283 (24.8%)	2625 (25.9%)	
	4	631 (15.6%)	161 (17.6%)	862 (16.7%)	1654 (16.3%)	
	5	361 (8.9%)	92 (10.0%)	600 (11.6%)	1053 (10.4%)	
	6	207 (5.1%)	60 (6.6%)	346 (6.7%)	613 (6.0%)	
	7	91 (2.3%)	22 (2.4%)	189 (3.7%)	302 (3.0%)	
	8	43 (1.1%)	11 (1.2%)	82 (1.6%)	136 (1.3%)	
	9	14 (0.3%)	7 (0.8%)	42 (0.8%)	63 (0.6%)	
	10	4 (0.1%)	2 (0.2%)	20 (0.4%)	26 (0.2%)	
	11	4 (0.1%)	1 (0.1%)	8 (0.2%)	13 (0.1%)	
	12	0 (0.0%)	0 (0.0%)	4 (0.1%)	4 (0.03%)	
	13	0 (0.0%)	0 (0.0%)	3 (0.1%)	3 (0.03%)	
Multimorbidity severity, n (%)	Common (2–3)	2685 (66.5%)	560 (61.1%)	3018 (58.3%)	6263(61.8%)	*<0.001*
	Severe (≥4)	1355 (33.5%)	356 (38.9%)	2156 (41.7%)	3867(38.2%)	

**Table 2:** Association Between Physical Activity and Severe Multimorbidity in the CHARLS Sample.

Physical Activity	Model 1 OR (95% CI) *p-Value*		Model 2 OR (95% CI) *p-Value*		Model 3 OR (95% CI) *p-Value*		Model 4 OR (95% CI) *p-Value*	
Inactive	1.00 (Ref)							
Active	0.89 (0.77–1.03)	*0.112*	0.94 (0.81–1.09)	*0.406*	0.94 (0.81–1.09)	*0.419*	1.06 (0.91–1.23)	*0.446*
Highly active	0.71 (0.65–0.77)	*<0.001*	0.80 (0.73–0.88)	*<0.001*	0.80 (0.73–0.88)	*<0.001*	0.89 (0.81–0.98)	*0.015*
N	10,130							

**Table 3:** Association Between Physical Activity and Severe Multimorbidity Stratified by Sex in CHARLS 2018.

Physical Activity	Women (N = 5540) OR (95% CI) *p-Value*		Men (N = 4590) OR (95% CI) *p-Value*	
Inactive	1.00 (Ref)			
Active	0.92 (0.76–1.11)	*0.409*	0.97(0.76–1.22)	*0.784*
Highly active	0.78 (0.69–0.88)	*<0.001*	0.83(0.73–0.95)	*0.006*

**Table 4:** Wald Tests for Interaction Between Physical Activity and Demographic Factors.

Group	χ^2^	df	*p* *-* *Value*
PA × Gender	0.38	2	*0.828*
PA × Age	7.39	2	*0.025*

**Table 5:** Association Between Physical Activity and Severe Multimorbidity Stratified by Age Group in CHARLS 2018.

Physical Activity	Age < 60 (N = 3596) OR (95% CI) *p-Value*		Age ≥ 60 (N = 6534) OR (95% CI) *p-Value*	
Inactive	1.00 (Ref)			
Active	1.11 (0.87–1.44)	*0.395*	0.85 (0.73–1.01)	*0.073*
Highly active	0.91 (0.78–1.06)	*0.213*	0.71 (0.64–0.80)	*<0.001*

**Table 6:** Analysis in the Supplementary Community Sample.

Physical Activity	Model 1 OR (95% CI) *p-Value*		Model 2 OR (95% CI) *p-Value*		Model 3 OR (95% CI) *p-Value*	
Inactive	1.00 (Ref)					
Active	0.73 (0.37–1.45)	*0.368*	0.77 (0.35–1.69)	*p* = 0.511	0.75 (0.34–1.68)	*0.490*
Highly active	0.28 (0.14–0.54)	*<0.001*	0.22 (0.10–0.51)	*<0.001*	0.21 (0.09–0.49)	*<0.001*
**N**	**315**					

**Table 7:** Performance Metrics of Machine Learning Models.

Dataset	Metric	LR	LightGBM	MLP	RF	SVM	XGBoost
Train	AUC	0.683	0.695	0.757	0.699	0.702	0.695
	Accuracy	66.15	66.98	71.48	67.43	67.97	67.25
	F1 Score	42.75	45.8	56.61	46.01	45.71	46.27
	Precision	60.34	61.35	67.54	62.68	64.77	61.92
	Sensitivity	33.1	36.53	48.73	36.35	35.32	36.94
	Specificity	86.56	85.79	85.54	86.63	88.14	85.97
Test	AUC	0.664	0.672	0.646	0.671	0.659	0.673
	Accuracy	66.12	66.38	64.21	66.18	66.12	66.12
	F1 Score	42.59	44.64	46.56	43.64	42.84	44.44
	Precision	60.25	60.06	54.11	59.94	60.12	59.37
	Sensitivity	32.93	35.52	40.86	34.31	33.28	35.52
	Specificity	86.6	85.43	78.62	85.85	86.38	85
Val	AUC	0.672	0.675	0.654	0.679	0.658	0.676
	Accuracy	65.2	65.39	65.13	65.92	65.13	65.39
	F1 Score	37.84	40.5	45.36	40.6	37.94	40.5
	Precision	59.41	58.88	56.41	60.62	59.12	58.88
	Sensitivity	27.76	30.86	37.93	30.52	27.93	30.86
	Specificity	88.3	86.7	81.91	87.77	88.09	86.7

## Data Availability

The CHARLS data that support the findings of this study are publicly available from the China Health and Retirement Longitudinal Study (CHARLS) repository, accessible at the official website: https://charls.pku.edu.cn, following registration and application approval. The community-based dataset generated and analyzed in this study is included within the article and its Supplementary Materials.

## Supplementary Materials

The following supporting information can be downloaded at: https://www.mdpi.com/article/10.3390/healthcare14152417/s1.

## Abbreviations

The following abbreviations are used in this manuscript:

PA	Physical Activity
MPA	Moderate Physical Activity
VPA	Vigorous Physical Activity
SHAP	SHapley Additive exPlanations
CHARLS	China Health and Retirement Longitudinal Study
ADL	Activities of Daily Living
SRH	Self-rated health
WHO	World Health Organization
OR	Odds Ratio
CI	Confidence Interval
Ref	Reference Category
AUC	Area Under the Curve
ROC	Receiver Operating Characteristic curve
