# Supplementary material for: Association of Physical Activity Levels with Severe Multimorbidity Among Middle-Aged and Older Adults: Evidence from CHARLS and a Community Sample
Source: Healthcare (Basel). 2026 Aug 5;14(15):2417. doi: 10.3390/healthcare14152417 (PMC13463940; doi:10.3390/healthcare14152417)
Supplement: Supplementary file 1 [file healthcare-14-02417-s001.zip › healthcare-4402034-supplementary.pdf]

Supplementary Materials

Table S1. Definition of the Outcome Variable for Multimorbidity Severity

Variable	Meaning	Values	Description
Multimorbidity severity	General multimorbidity	0	2–3 chronic diseases
	Severe multimorbidity	1	4 or more chronic diseases

Table S2. Construction and Definition of Physical Activity Level Variables

Variable	Meaning	Values	Description
Mod days	Days/week of moderate-intensity physical activity	Numeric	Number of days per week on which the participant engaged in moderate-intensity physical activity.
Mod gt30	Session duration of moderate-intensity physical activity >30 min	0 = No; 1 = Yes	Whether each session of moderate-intensity physical activity lasted more than 30 minutes.
Mod gt120	Session duration of moderate-intensity physical activity >120 min	0 = No; 1 = Yes	Whether each session of moderate-intensity physical activity lasted more than 120 minutes.
Mod gt240	Session duration of moderate-intensity physical activity >240 min	0 = No; 1 = Yes	Whether each session of moderate-intensity physical activity lasted more than 240 minutes.
Int days	Days/week of vigorous-intensity physical activity	Numeric	Number of days per week on which the participant engaged in vigorous-intensity physical activity.
Int gt30	Session duration of vigorous-intensity physical activity >30 min	0 = No; 1 = Yes	Whether each session of vigorous-intensity physical activity lasted more than 30 minutes.
Int gt120	Session duration of vigorous-intensity physical activity >120 min	0 = No; 1 = Yes	Whether each session of vigorous-intensity physical activity lasted more than 120 minutes.
Int gt240	Session duration of vigorous-intensity physical activity >240 min	0 = No; 1 = Yes	Whether each session of vigorous-intensity physical activity lasted more than 240 minutes.
Mpa meet	Moderate-intensity physical activity meeting the minimum recommended level	0 = No; 1 = Yes	Rule-based indicator derived from weekly frequency and session-duration categories. Defined as 1 if any of the following criteria were met: Mod days ≥ 5 and Mod gt30 = 1; or Mod days ≥ 3 and Mod gt120 = 1; or Mod days ≥ 2 and Mod gt240 = 1. This variable was used to identify individuals whose moderate-intensity activity reached a level corresponding to the WHO minimum recommendation (≥150 min/week).

Vpa meet	Vigorous-intensity physical activity meeting the minimum recommended level	0 = No; 1 = Yes	Rule-based indicator derived from weekly frequency and session-duration categories. Defined as 1 if any of the following criteria were met: Int days ≥ 3 and Int gt30 = 1; or Int days ≥ 1 and Int gt120 = 1; or Int days ≥ 1 and Int gt240 = 1. This variable was used to identify individuals whose vigorous-intensity activity reached a level corresponding to the WHO minimum recommendation (≥75 min/week).
High mpa	Moderate-intensity physical activity at the highly active level	0 = No; 1 = Yes	Rule-based indicator derived from weekly frequency and session-duration categories. Defined as 1 if any of the following criteria were met: Mod days ≥ 5 and Mod gt120 = 1; or Mod days ≥ 3 and Mod gt240 = 1. This variable was used to identify individuals whose moderate-intensity activity reached a level corresponding to the WHO range associated with additional health benefits (>300 min/week).
High vpa	Vigorous-intensity physical activity at the highly active level	0 = No; 1 = Yes	Rule-based indicator derived from weekly frequency and session-duration categories. Defined as 1 if any of the following criteria were met: Int days ≥ 5 and Int gt30 = 1; or Int days ≥ 2 and Int gt120 = 1; or Int days ≥ 1 and Int gt240 = 1. This variable was used to identify individuals whose vigorous-intensity activity reached a level corresponding to the WHO range associated with additional health benefits (>150 min/week).
PA cat	Three-level physical activity category	0 = Inactive; 1 = Active; 2 = Highly active	Mutually exclusive hierarchical classification. Participants were first classified as highly active if High mpa = 1 or High vpa = 1; among the remaining participants, those with Mpa meet = 1 or Vpa meet = 1 were classified as active; all others were classified as inactive.

Table S3. Physical Activity Level Questionnaire Items and Classification Criteria

Question:

In the past 7 days, have you engaged in any physical activity that made your body warm, caused sweating, or increased your breathing? Examples include brisk walking, square dancing, cycling, aerobics, stair climbing, tai chi, lifting heavy objects, or jogging.

Please select the one that best describes your actual situation. If more than one option applies, choose the one that represents the highest level of activity.

Category	Criteria	Values
Inactive	Little to no moderate- or vigorous-intensity physical activity lasting more than 10 minutes per week; or mainly light activities such as easy walking, general housework, cooking, or laundry, not meeting the minimum recommended level.	0

Active	Meeting the basic recommended level per week but not reaching the highly active level. Meets any of the following: (a) At least 150 but less than 300 minutes of moderate-intensity physical activity per week (e.g., about 3–5 days per week, 30–50 minutes each session, totaling >150 minutes per week); (b) At least 75 but less than 150 minutes of vigorous-intensity physical activity per week (e.g., about 3 days per week, 25–40 minutes each session, or totaling >1 hour 15 minutes but <2 hours 30 minutes per week).	1
Highly active:	Engaging in longer duration or higher intensity physical activity per week, reaching a high activity level. Meets any of the following: (a) At least 300 minutes of moderate-intensity physical activity per week (e.g., about 5–6 days per week, 50–60 minutes each session, or totaling ⩾5 hours per week); (b) At least 150 minutes of vigorous-intensity physical activity per week (e.g., about 3 days per week, 50 minutes each session, or about 4 days per week, 40 minutes each session).	2

Intensity definitions: Moderate-intensity physical activity: Activities that make the body warm, cause slight sweating, and increase breathing rate compared to rest, but still allow normal conversation (e.g., brisk walking, square dancing, cycling, aerobics, stair climbing, general farm work, heavier housework). Vigorous-intensity physical activity: Activities that cause noticeable sweating, markedly increased heart rate and breathing, and make speaking difficult (e.g., jogging, faster cycling, steep uphill climbing, lifting heavy objects, heavy manual farm work).

Table S4. Definitions and Coding of Covariates

Variable	Category	Values
Age	Continuous variable, years	Mean ± SD
Sex	Female	0
	Male	1
Marital status	No	0
	Yes	1
Education	Primary school or below	1
	Junior high school	2
	Senior high / vocational	3
	College or above	4
Drinking status	NO	0
	YES	1
Smoking status	NO	0
	YES	1
SRH	Very poor	1
	Poor	2
	Fair	3
	Good	4

	Very good	5
ADL impairment	Number of impaired ADL items	0-6

Table S5. Association Between Physical Activity and Severe Multimorbidity (⩾4 Chronic Conditions) in the Complete Eligible CHARLS Population

Physical	Model 1		Model 2		Model 3		Model 4	
activity	OR(95% CI)	*P-value*	OR(95% CI)	*P-value*	OR(95% CI)	P-value	OR(95% CI)	*P-value*
Inactive	1.00 (Ref)							
Active	0.83 (0.73–0.95)	*0.005*	0.90 (0.79–1.03)	*0.114*	0.90 (0.79–1.03)	0.123	1.04 (0.91–1.20)	*0.562*
Highly active	0.63 (0.59–0.68)	*<0.001*	0.77 (0.71–0.83)	*<0.001*	0.77 (0.71–0.83)	<0.001	0.87 (0.79–0.94)	*0.001*
N	17783							

Table S6. WHO-Equivalent Physical Activity Reclassification and Sensitivity Analysis in the CHARLS Sample

A. Construction and Definition of WHO-Equivalent Physical Activity Categories

Variable	Meaning	Values	Description
Mod min/day sens	Assigned duration of moderate-intensity physical activity per activity day	0, 30, 120, or 240 min	Assigned according to the highest reported duration threshold for moderate-intensity physical activity: no reported duration threshold = 0 min; Mod gt30 = 1 = 30 min; Mod gt120 = 1 = 120 min; and Mod gt240 = 1 = 240 min. If more than one duration threshold was reported, the longest corresponding duration was used. Participants reporting no moderate-intensity physical activity were assigned 0 min.
Vig min/day sens	Assigned duration of vigorous-intensity physical activity per activity day	0, 30, 120, or 240 min	Assigned according to the highest reported duration threshold for vigorous-intensity physical activity: no reported duration threshold = 0 min; Int gt30 = 1 = 30 min; Int gt120 = 1 = 120 min; and Int gt240 = 1 = 240 min. If more than one duration threshold was reported, the longest corresponding duration was used. Participants reporting no vigorous-intensity physical activity were assigned 0 min.
Mod week min sens	Weekly moderate-intensity physical activity volume	Numeric, min/week	Calculated as Mod days × Mod min/day sens.
Vig week min sens	Weekly vigorous-intensity	Numeric, min/week	Calculated as Int days × Vig min/day sens.

	physical activity volume		
MVPA equivalent min sens	WHO-equivalent weekly physical activity volume	Numeric, moderate-intensity-equivalent min/week	Calculated as Mod week min sens + 2 × Vig week min sens, with 1 min of vigorous-intensity physical activity treated as equivalent to 2 min of moderate-intensity physical activity.
PA cat WHO sens	WHO-equivalent three-level physical activity category	0 = Inactive; 1 = Active; 2 = Highly active	Mutually exclusive classification based on total WHO-equivalent weekly physical activity volume: inactive, <150 equivalent min/week; active, 150 to <300 equivalent min/week; and highly active, ≥300 equivalent min/week. Thus, values exactly equal to 150 min/week were classified as active, whereas values exactly equal to 300 min/week were classified as highly active.

B. Association Between WHO-Equivalent Physical Activity and Severe Multimorbidity in the CHARLS Sample

Physical	Model 1		Model 2		Model 3		Model 4	
activity	OR(95% CI)	*P-value*	OR(95% CI)	*P-value*	OR(95% CI)	*P-value*	OR(95% CI)	*P-value*
Inactive	1.00 (Ref)							
Active	0.89 (0.77–1.03)	*0.113*	0.94 (0.81–1.09)	*0.409*	0.94 (0.81–1.09)	*0.422*	1.06 (0.91–1.23)	*0.443*
Highly active	0.71 (0.65–0.77)	*<0.001*	0.80 (0.73–0.88)	*<0.001*	0.80 (0.73–0.88)	*<0.001*	0.89 (0.81–0.98)	*0.016*
N	10130							

Table S7. Association Between Physical Activity and Severe Multimorbidity in the CHARLS Sample, Redefined Using the Seven Chronic Diseases Common to Both Samples

Physical	Model 1		Model 2		Model 3		Model 4	
activity	OR(95% CI)	*P-value*	OR(95% CI)	*P-value*	OR(95% CI)	*P-value*	OR(95% CI)	*P-value*
Inactive	1.00 (Ref)							
Active	0.74 (0.58–0.96)	*0.022*	0.77 (0.59–0.99)	*0.042*	0.78 (0.60–1.01)	*0.057*	0.89 (0.69–1.16)	*0.397*
Highly active	0.59 (0.51–0.70)	*<0.001*	0.64 (0.54–0.76)	*<0.001*	0.65 (0.55–0.77)	*<0.001*	0.73 (0.61–0.87)	*<0.001*
N	5249							

Table S8. Baseline Characteristics of Participants in the Supplementary Community Sample

Variable	Category	Highly active (N=165)	Active (N=67)	Inactive (N=83)	Total (N=315)	*P-value*
Age, Mean ± SD		69.02 ± 6.36	78.31 ±9.39	70.87 ±7.00	71.48 ±8.11	*<0.001*

Gender,n (%)	Female	133 (80.6%)	43 (64.2%)	50 (60.2%)	226 (71.7%)	*0.001*
	Male	32 (19.4%)	24 (35.8%)	33 (39.8%)	89 (28.3%)	
Marital status,n (%)	No	123 (74.5%)	56 (83.6%)	63 (75.9%)	242 (76.8%)	*0.326*
	Yes	42 (25.5%)	11 (16.4%)	20 (24.1%)	73 (23.2%)	
Drinking,n (%)	NO	128 (77.6%)	52 (77.6%)	53 (63.9%)	233 (74.0%)	*0.050*
	YES	37 (22.4%)	15 (22.4%)	30 (36.1%)	82 (26.0%)	
Smoking,n (%)	NO	139 (84.2%)	54 (80.6%)	57 (68.7%)	250 (79.4%)	*0.016*
	YES	26 (15.8%)	13 (19.4%)	26 (31.3%)	65 (20.6%)	
Education,n (%)	Primary school or below	69 (41.8%)	49 (73.1%)	48 (57.8%)	166 (52.7%)	*<0.001*
	Junior/senior high school or vocational school	59 (35.8%)	7 (10.4%)	21 (25.3%)	87 (27.6%)	
	College or above	37 (22.4%)	11 (16.4%)	14 (16.9%)	62 (19.7%)	
Multimorbidity Count,n (%)	2	112 (67.9%)	20 (29.9%)	47 (56.6%)	179 (56.8%)	*<0.001*
	3	31 (18.8%)	23 (34.3%)	12 (14.5%)	66 (21.0%)	
	4	18 (10.9%)	16 (23.9%)	17 (20.5%)	51 (16.2%)	
	5	4 (2.4%)	8 (11.9%)	6 (7.2%)	18 (5.7%)	
	6	0 (0.0%)	0 (0.0%)	1 (1.2%)	1 (0.3%)	
Multimorbidity severity,n (%)	Common (2-3)	143 (86.7%)	43 (64.2%)	59 (71.1%)	245 (77.8%)	*<0.001*
	Severe (≥4)	22 (13.3%)	24 (35.8%)	24 (28.9%)	70 (22.2%)	
